# Retraction: Beyond emotional and spatial processes: Cognitive dysfunction in a depressive phenotype produced by long photoperiod exposure

**DOI:** 10.1371/journal.pone.0339231

**Published:** 2025-12-19

**Authors:** The PLOS One Editors

Following publication of this article [1], the University of Tennessee, Knoxville (UT Knoxville) notified PLOS that an institutional investigation concluded that research misconduct occurred. Specifically, PLOS was informed that the UT Knoxville Investigation Committee found falsification of research results and that the reported study did not have appropriate animal ethics approval as follows:

- Animal data were inappropriately excluded from the analysis reported in the article [1]; the Investigation Committee concluded that the corresponding author instructed the student authors not to report information about the exclusion of animal data in the preparation of this article [1].
- The animal ethics approval from the University of Tennessee Animal Care Committee, cited in this article [1] as Protocol Number: 2349-UTK, did not cover all behavioral tests reported. The Investigation Committee stated that behavioral tests including novel object recognition (NOR), elevated plus maze, and forced swim test were not included in the original study protocol and approval for an amendment to the protocol to include these additional experiments was obtained on July 1, 2016. However, experimental records indicate that these tests were conducted in December 2015 and January 2016, prior to obtaining an updated approval.
- The use of the long photoperiod experiments was not approved under the ethics approval 2349-UTK cited in this article [1], but was approved at a later date on May 1, 2018, under a different approval number, 2565.

Additionally, PLOS noted that the individual-level quantitative data underlying Figs 2-4 are not provided within this article [1] as stated in the article’s Data Availability statement. The authors did not respond to editorial queries about these concerns.

The above issues raise concerns about adherence to journal policies on data availability and ethical oversight and about the validity and integrity of the reported results and conclusions. In light of these issues, and in accordance with the request of The University of Tennessee, Knoxville, the *PLOS One* Editors retract this article.

All authors either did not respond directly or could not be reached.
